# An Environmentally Friendly Flow Injection-Gas Diffusion System Using Roselle (*Hibiscus sabdariffa* L.) Extract as Natural Reagent for the Photometric Determination of Sulfite in Wines

**DOI:** 10.1155/2021/6665848

**Published:** 2021-05-18

**Authors:** Preeyaporn Reanpang, Teerarat Pun-uam, Jaroon Jakmunee, Supada Khonyoung

**Affiliations:** ^1^Department of Chemistry, Faculty of Science and Technology, Thammasat University, Lampang 52190, Thailand; ^2^Department of Chemistry, Faculty of Science and Technology, Thammasat University, Rangsit, Pathumthani 12120, Thailand; ^3^Department of Chemistry and Center of Excellence for Innovation in Chemistry, Faculty of Science, Chiang Mai University, Chiang Mai 50200, Thailand; ^4^Research Center on Chemistry for Development of Health Promoting Products from Northern Resources, Faculty of Science, Chiang Mai University, Chiang Mai 50200, Thailand

## Abstract

In this work, a green and simpler method for photometric determination of sulfite based on a flow injection-gas diffusion (FI-GD) system using a natural reagent extracted from roselle (*Hibiscus sabdariffa* L.) was proposed. Despite the fact that the employed reaction is not selective to sulfite, its sensitivity is high, and the selectivity can be improved by coupling a GD unit to the FI system. The method involves monitoring a decrease in absorbance of the reagent solution that is used as an acceptor solution. When a standard solution or sample solution was injected into an acidic donor stream, the liberated sulfur dioxide diffuses through a gas-permeable membrane of the GD unit into the acceptor solution, causing color fading of the reagent. A linear analytical curve in the range of 5–100 mg L^−1^ was obtained with a detection limit of 2 mg·L^−1^. Relative standard deviations of 0.9%, 0.6%, and 0.6% were obtained for the determination of 30, 70, and 100 mg·L^−1^ SO_3_^2-^ (*n* = 11). The developed method was applied to wine samples, giving results that agreed with those obtained with the Ripper titrimetric method. The proposed method offers advantages of simplicity, cost-effectiveness, and being environmentally friendly such as reduced chemical consumption and less waste generation.

## 1. Introduction

Sulfite is widely used in food and beverage industries as a preservative to inhibit microbial activity and to control enzymatic reactions during the fermentation and storage stages. Although sulfite is often used as food additives, contents above the permissible limit may lead to adverse effects to the consumers, such as nasal congestion, coughing, breathing difficulties, asthma, itching, and other skin rashes [1]. The control and regulation of the use of sulfites in food and beverages are very important. The US Food and Drug Administration (FDA) recommends that there should be warning labels on any food containing more than 10 mg·kg^−1^ sulfite or on any beverage containing more than 10 mg·L^−1^ sulfite [2].

There are several analytical methods for sulfite determination in food and beverages, including the Monier-Williams method [3], the Ripper method (iodometric titration) [4], and ion exchange chromatographic method [5]. Most of these have limitations on slow and laborious procedures, consuming large amounts of chemicals and requiring expensive equipment. Previously, electrochemical approaches [6–9] have been reported for sulfite determination to reduce the amount of chemical reagent used, but it involves a variety of instruments and complicated modification steps such as cleaning of the electrode surface and immobilizing various species on the electrode surface (include chemisorption, covalent binding, electropolymerization, and others). Thus, UV-vis spectrophotometry is still an interesting technique for routine application due to widely available instrumentation and simple straightforward procedures. Several chromogenic reagents have been selected for the sulfite spectrophotometric determination such as *o*-phthaldialdehyde [10], diaquacobyrinic acid heptamethyl ester [11], and pararosaniline [12]. However, the use of synthetic chemicals as reagents has a great disadvantage because the reagent itself is more toxic than the analyte. The amounts of reagents used in some techniques are also high, leading to the generation of large quantities of hazardous waste. Therefore, alternative environmentally friendly chemicals derived from natural resources such as plant extracts are attracting more attention. This supports the green analytical chemistry concept that encourages utilizing chemicals from natural products instead of toxic synthetic compounds in chemical analysis and reducing amounts of waste released to the environment. In addition, the use of natural reagent contributes to increasing the safety of the operator. Recently, use of natural reagents in combination with flow-based analytical systems was proposed to achieve a greener analytical method, for example, using reagents derived from pumpkin [13], *Morinda citrifolia* root [14], heartwood [15], *Phyllanthus emblica* Linn [16], peacock flower [17], and orchid flower [18] for determination of various analytes. For sulfite determination, a crude extract of sweet potato root (*Ipomoea batatas* (L.) Lam.) [19] and *Tibouchina granulosa* flowers [20] were utilized as natural reagents.

Bleaching reactions of anthocyanins by sulfites have been well known for many years. The addition of sulfite in the C-2 or C-4 position of flavilic ring and blocking of electronic delocalization in anthocyanin molecule results in color fading of the solution to become colorless [21, 22]. Anthocyanin could be found in plants, especially flowers and fruits. Roselle (*Hibiscus sabdariffa* L.) is a good source of anthocyanin [23], often used as food, herbal, drink, hot, or cold beverages, and flavoring agent in the food industry. In Thailand, instant dried roselle powder is commonly used for making tea or juice and can be easily found in local markets.

A flow injection analysis can provide rapid, high precision, high accuracy, lower amount of reagents and solvents, and automated method for sulfite determination [24–27]. The selectivity of the method was increased by inserting a GD unit into FI system [10, 28, 29]. The sample solution was aspirated into a donor channel of GD unit. Sulfite was separated from sample matrices through the membrane into an acceptor channel and merged in line with a continuously flowing reagent solution prior to subsequent monitoring by a detector placing after the GD unit on the acceptor side. The GD incorporated with FI analysis can eliminate interferences in sample such as colored substances and colloidal particles.

In this work, we proposed the environmentally friendly method for the determination of sulfite by exploiting roselle extract as a natural reagent in a simple FI-GD photometric system. A crude extract of spray-dried roselle powder was used as a reagent in an acceptor solution of the FI-GD system. Sulfur dioxide that is converted from sulfite in an acidic donor stream diffuses through a PTFE membrane of the GD unit to dissolve in an acceptor stream. It reacts with anthocyanin contained in the natural reagent, leading to the bleaching of the reagent color, which is monitored by a home-made photometer. The conditions for operating the developed system were optimized. The proposed method was applied for the determination of sulfite in various types of wine samples.

## 2. Experimental

### 2.1. Chemicals

All chemicals used were of analytical reagent grade. Deionized water (obtained from a water purification system of Elgstat Option 3A, Elga, England) was used throughout. A stock standard solution (1000 mg·L^−1^) was prepared by dissolving 0.1575 g of Na_2_SO_3_ (Sigma Aldrich, USA) in 1.0% (v/v) ethanol (RCI Labscan, Thailand) and filling the volume up to 100 mL with this ethanol solution. Working standard solutions were daily made by dilutions of the stock one in 1.0% (v/v) ethanol. Acetate buffer solution pH 3 (0.1 mol L^−1^) was prepared by mixing 982.3 mL of 0.1 mol·L^−1^ acetic acid (Merck, Germany) with 17.7 mL of 0.1 mol·L^−1^ CH_3_COONa∙3H_2_O (BDH, England). The solutions used for the Ripper method were prepared according to the reference method [30].

### 2.2. Preparation of Natural Reagent

The roselle reagent was daily prepared by extracting 1.4 g of the spray-dried roselle powder (purchased from Thiptipa Company Ltd., Thailand) with 100 mL of acetate buffer solution pH 3.0. The suspension was then centrifuged at 4,000 rpm for 10 minutes to obtain a clear solution, filtered through a Whatman #1 filter paper, and the volume was then made up to 100 mL. To confirm the extraction reproducibility of different batches of roselle reagent, the absorbance of the obtained solution was measured before use. The acceptable absorbance of the extracted solution was 1.525 ± 0.025 at 510 nm. The content of total anthocyanin in the roselle solution determined by pH differential method [31] was 15.2 mg per g of the roselle powder.

### 2.3. FI-GD Photometric System

**Figure 1:**
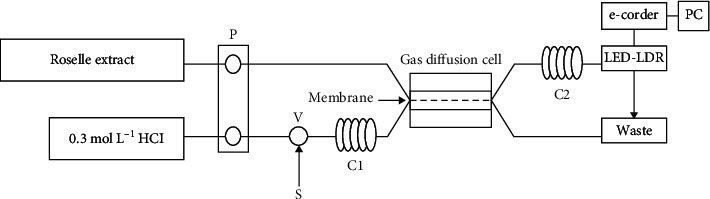
Manifold of FI-GD photometric flow system for determination of sulfite; P = peristatic pump, V = six-port valve, S = standard/sample, C1 and C2 = mixing coils, and PC = personal computer.

The FI-GD photometric analyzer (Figure 1) consists of a peristaltic pump (Ismatec, Switzerland) fitted with Tygon pump tubings 1.14 mm i.d., PTFE tubing 0.7 mm i.d. (Ismatec, Switzerland), a six-port valve (FLOM, Japan) with a 100 *μ*L sample loop, a home-made GD unit, a simple home-made light-emitting diode-light-dependent resistor (LED-LDR) photometer [32] using a green LED (maximum emission: 520 nm) as a light source, equipped with 10 mm path length flow-through cell (Hellma, Germany), an e-corder (eDAQ, Australia) as a data acquisition unit, and a personal computer.

The GD unit was made of two acrylic blocks (160 mm long, 50 mm wide, and 15 mm thick), engraved by a CO_2_ laser cutting machine (CNC Bro, China) for donor and acceptor channels (each 300 mm long, 1.5 mm wide, and 0.5 mm deep), as depicted in Figure S1 (supplementary information). A polytetrafluoroethylene (PTFE) membrane (plumber tape) was sandwiched between the two blocks to form channels for donor and acceptor solutions to flow on each side. A commercial PTFE plumber tape (i.d. 0.7 mm) (Thai Pipe JORE-TEX, Thailand) available in a local market was employed as a gas diffusion membrane. This membrane, inexpensive and durable, could be used for more than 200 injections without apparent variations.

### 2.4. FI-GD Procedure

A standard/sample solution was injected manually into a 0.3 mol·L^−1^ HCl donor stream and flowed through a mixing coil (C1) to a GD unit, where any sulfite present was converted to sulfur dioxide and diffused through a PTFE membrane into an acceptor stream of the roselle reagent. Sulfur dioxide reduced the color intensity of the reagent, which was monitored by measuring a fading of the color at the flow-through cell. The output signal from the detector was recorded as a peak on a personal computer. Peak height was directly proportional to the concentration of sulfite in the injected solution. An analytical curve was constructed by plotting peak height versus sulfite concentration.

### 2.5. Determination of Sulfite by the Ripper Method

An accurate sample volume of 10.00 mL was transferred to an Erlenmeyer flask; an aliquot of 5.00 mL of 1% w/v starch indicator and a pinch of sodium hydrogen carbonate were added. After that, 5.00 mL of 33% (v/v) sulfuric acid was added, and the solution was immediately titrated with an 0.25 mmol·L^−1^ iodine solution to a blue endpoint (color stable for 20 seconds) [30].

### 2.6. Sample Preparation

White, red, and sparkling wines were purchased from local markets in Bangkok, Thailand. The samples were poured into a beaker and degassed for 20–30 minutes before analysis by the Ripper method and the proposed system.

## 3. Results and Discussion

### 3.1. A Preliminary Study of the Reaction

**Figure 2:**
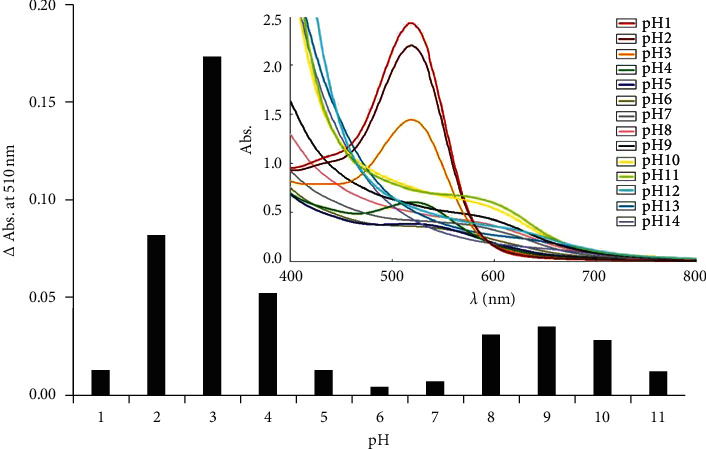
Influence of pH on ΔAbs. of roselle extract. Inset: spectra of roselle extract in various pH.

Anthocyanin undergoes structure transformations with a change in the pH, which has a dramatic effect on its color. The bleaching reaction of anthocyanin by sulfite was investigated under different pH values (1.0 to 14.0), adjusting by NaOH or HCl solutions. Solutions containing 100 mg·L^−1^ sulfite and 1.0% (w/v) of roselle extract in different pH media were scanned for absorption spectra, using correspondent reagent blanks at each pH as references. The difference of absorbances (at 510.0 nm) of roselle extract without and with the added standard solution is shown in Figure 2. It is found that pH 3.0 gave the highest absorbance difference as compared to the other pH values. At pH values of 3.0 or lower, the flavylium cation (pKa is 1–3) is the predominant, which has an orange to red color. At increasing pH, the flavylium cation changes rapidly to form the colorless carbinol pseudobase followed by the formation of quinonoid monoanions (purple color). The pKa of quinonoid monoanions are 7.5–8.0 [21], resulting in the slightly increased absorbance at pH values near 9.0. The chromophore of eight conjugated double bonds carrying a positive charge on the heterocyclic oxygen ring is responsible for the red color produced by anthocyanins under acidic conditions pH 3. Adding sulfite at C-2 or C-4 position of the flavilic ring results in disruption of conjugation in the anthocyanin molecule causing bleaching of its color [22]. Therefore, the pH of the solution was fixed at 3.0 in further experiments by using an acetate buffer solution.

### 3.2. Optimization of FI-GD Photometric System

The optimization of the FI-GD analyzer was performed by using the following conditions: 0.3 mol·L^−1^ HCl as a carrier stream for donor solution, 30 cm mixing coil length for mixing between sample/standard and the HCl solution, and 100 cm mixing coil length for mixing between the diffused SO_2_ and roselle extract reagent. Effects of roselle extract concentration, types of PTFE plumber tape, the effect of the flow rate of acceptor and donor stream, and effect of the sample volume were investigated.

**Figure 3:**
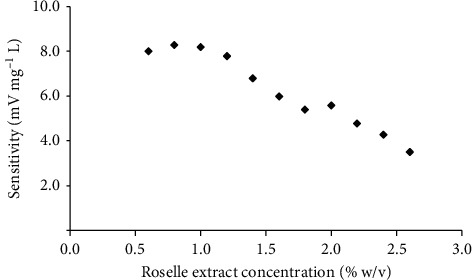
Effect of reagent concentration (% w/v) on sensitivity of sulfite determination.

Various roselle extract concentrations were studied in the range of 0.6–2.6% (w/v). Sulfite standard solutions in the concentration range of 5–100 mg·L^−1^ were injected into the system to construct an analytical curve for each reagent concentration. The analytical curve was made by plotting peak height (difference between signals without and with analyte presence) obtained versus sulfite concentration. It was found that the sensitivity (slope of graph) decreased with the increase of reagent concentrations (Figure 3). At high reagent concentrations (1.6–2.6% (w/v)), the low sensitivity was obtained because of a high color intensity of the reagent extract. When the standard solution was inserted, generated SO_2_ diffused through membrane and the color intensity of the roselle extract-sulfite mixture was decreased, but there is a small change of color intensity from the baseline level. In contrast, a lower reagent concentration (0.6–1.2% (w/v)) provided higher sensitivity, but the linearity was decreased (Table S1) because the amount of anthocyanin in the reagent was not enough to react with a high concentration of sulfite. Therefore, 1.4% (w/v) of reagent was selected as a suitable condition because this concentration has an excess amount of anthocyanin in the reagent that provided high sensitivity and good linearity.

**Figure 4:**
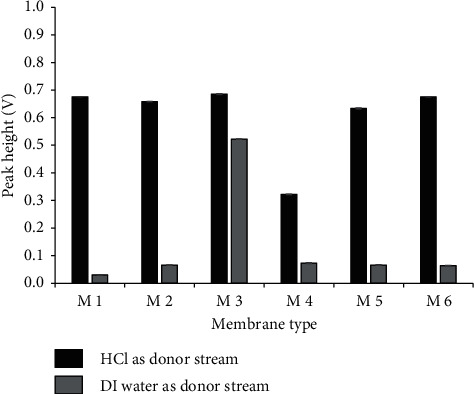
Influence of the membrane types on peak height of 100 mg·L^−1^ sulfite when using HCl or DI water as a donor stream.

Six commercial PTFE plumber tapes (represented by M1 to M6, Figure 4) were used as a membrane for the separation of SO_2_ gas from the donor stream to the acceptor stream. The hydrophobic membrane should allow only gas species to pass through it effectively, so high selectivity and sensitivity would be obtained. Experiments were carried out in two cases, using 0.3 mol·L^−1^ HCl and water as donor solutions; the 100 mg·L^−1^ sulfite standard solution was injected into the donor stream and the FI-peak resulting from color fading of the reagent in the acceptor stream was recorded. The peak heights of 100 mg·L^−1^ sulfite standard solution using HCl and DI water as the donor stream were compared as shown in Figure 4. It is observed that the membrane M1 (plumber tape from Thai Pipe JORE-TEX company) provided the highest peak height difference. The membrane M3 gave a dramatic leaking of sulfite ion from the donor stream to the acceptor stream. A suitable membrane must allow permeation of only gaseous SO_2_, while the ionic SO_3_^2−^ form should not be transported. Thus, the M1 membrane was selected for further experiments.

Influence of the acceptor flow rates was studied in the range of 0.5–2.0 mL·min^−1^ by fixing the flow rate of the donor stream at 2.0 mL·min^−1^. The result was presented in Table S2. The sensitivity decreased with the increase in the flow rate of the acceptor line. However, decreasing the flow rate led to the large dispersion of the sample zone and gave the lower linearity and less sample throughput as well. Thus, the acceptor flow rate at 1.0 mL·min^−1^ was selected due to the fact that this condition provided high sensitivity, good linearity, and high sample throughput.

The effect of donor flow was also examined in the range of 0.5–2.0 mL·min^−1^, which was related to the acceptor flow rate. From Table S3, it was found that the donor flow rate at 1.0 mL·min^−1^ was chosen. By employing the equal flow rates of donor and acceptor streams, there was no pressure difference on both sides of the membrane of the gas diffusion unit. This equal pressure results in the prevention of membrane damage.

Sample volume was optimized in the range of 50–300 *μ*L, as shown in Figure S2. The result showed that the sensitivity increased when a large amount of sample was injected into the system. However, increasing the injected sample volume causes poor precision and band broadening and leads to a loss in sampling frequency. Therefore, a sample volume of 100 *μ*L was selected.

### 3.3. Analytical Characteristics

Using the optimal conditions, roselle extract 1.4% (w/v) in acetate buffer pH 3 as an acceptor stream and 0.3 mol·L^−1^ HCl as a carrier stream for donor solution, flow rate of each stream of 1.0 mL·min^−1^, the length of mixing coil 1 and mixing coil 2 of 30 and 100 cm, respectively, and a sample volume of 100 *μ*L, analytical characteristics of the system were investigated. An analytical curve was linear in the range of 5–100 mg·L^−1^ (*y* = 0.0074x − 0.0068, *R*^2^ = 0.9993, and *n* = 6). A limit of detection of 2 mg·L^−1^ was achieved by injecting sulfite standard at the lowest concentration that can observe the signal different from the baseline. The precision of the method was evaluated by injecting standard solutions: 30, 70, and 100 mg·L^−1^ for 11 replicates, giving relative standard deviation percentages (%RSD) of the peak height of 0.9, 0.6, and 0.6, respectively. Furthermore, the stability of the proposed system and lifetime of the membrane were tested by continuously injecting a series of sulfite standards for 10 hours (210 injections). Analytical curves were constructed, and variation in slopes of 10 analytical curves was calculated as %RSD, which is found to be 2.1, indicating that the system is highly stable. The sampling frequency was 21 h^−1^, and the consumptions of 0.3 mol·L^−1^ HCl and extracted reagent per determination were about 2.9 mL.  Table 1 compares analytical characteristics of the proposed method with those of the previous procedures for photometric determinations of sulfite using various natural reagents.

The effects of different interfering compounds on the analytical signal of 10, 50, and 100 mg·L^−1^ standard solution were investigated. The tolerance limit of the interfering compounds was evaluated based on the recovery percentages within the range of 95–105%. All of the interfering compounds did not affect the percentage recovery except ethanol and ascorbic acid (Table 2). The ethanol molecule is able to pass through the membrane [32] and affects the degradation of anthocyanin [33]. Ethanol at a concentration of 30% v/v or higher affected recovery. In fact, the percentage of ethanol in wine was 7–24% [10], so ethanol did not interfere in the determination of sulfite in wine by this method. Additionally, the interfering effect was noted for ascorbic acid in concentration of 500 mg·L^−1^ or higher. Ascorbic acid is a reducing agent which is an electron donor for reducing sulfite to hydrogen sulfide [34]. However, the maximum level of ascorbic acid in wine was 150 mg·L^−1^ [35]. Thus, ascorbic acid did not cause any interfering effect.

### 3.4. Application to Real Samples

**Figure 5:**
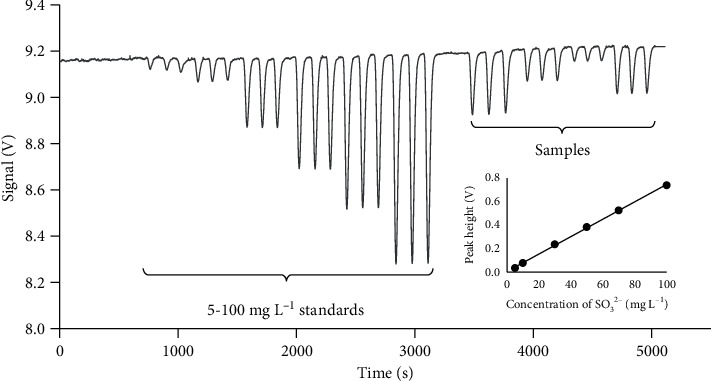
Recorder output of a routine run. Number of replicates: 3; left: standard solutions, right: sample; inset: plot of the analytical curve.

The determination of free sulfite was carried out in the various wine samples. Wine samples were prepared as described in Section 2.6 and diluted with deionized water (if necessary) and injected into the developed system. The FIAgrams of sulfite standard solutions and wine samples are shown in Figure 5. The same samples were analyzed by the Ripper titrimetric method for comparison, giving the results in Table 3. It is found that the results from the proposed method agreed with those from the Ripper method. The results from both methods were compared by *t*-test at 95% confidence level that indicates no significant difference between both methods (*t*_calculated_ = 1.91, *t*_critical_ = 2.23). The recovery test was performed to evaluate the influence of the sample matrix. The recovery values are between 95 and 105% for sparkling and white wines and 53–77% for red wine. The lower recovery percentage in the case of red wine may be due to the reaction of anthocyanin contained in red wine with sulfite that presented at a higher concentration than originally presented in the wine. The spiked sulfite may affect the equilibrium between anthocyanin and sulfite originally presented in the wine. From the result in Table 3, the RSD of the Ripper titrimetric method was found to be less than ±5%, showing high precision of the carefully performed titration. However, the direct titration of a sample without sample preparation can lead to poor accuracy if there are some interferences that can react with the titrant presented in the sample. In addition, the method can also be applied for total sulfite with some modification. The sample must be treated with NaOH to release the bound sulfite from the wine matrix.

## 4. Conclusion

The simple flow injection-gas diffusion photometric system was developed using roselle extract, a natural reagent, for the determination of sulfite. The developed method is an environmentally friendly and green analytical method and offered high selectivity by using a commercial PTFE plumber tape as a gas-permeable membrane in the GD unit. Moreover, a low-cost laboratory-made photometer provided good sensitivity and high sample throughput for the quantitation of sulfite in real samples. The results obtained from the proposed method and the Ripper method are in good agreement as compared by paired *t*-test at 95% confidence level. The proposed method was successfully applied to determine sulfite in wine samples, with advantages of simplicity, low cost, being environmentally friendly and with reduction of chemicals and thus toxic waste generation.

## Figures and Tables

**Table 1:** The analytical performances of spectrometric determination of sulfite using various natural reagents.

Crude extract	Reagent	Method	Linear range (mg L^−1^)	LOD (mg L^−1^)	Sample throughput (h^−1^)	Samples	Ref.
Sweet potato root (*Ipomoea batatas* (L.) Lam.)	Polyphenol oxidase	FI	3.2–48	0.18	26	White wine, white vinegar, juice	[19]
*Tibouchina granulosa* flowers	Anthocyanin	Batch	2–10	—	—	White wine	[20]
Roselle (*Hibiscus sabdariffa* L.)	Anthocyanin	FI-GD	5–100	2	21	Sparkling wine, white wine, red wine	This work

**Table 2:** Effect of some potential interfering species.

Potential interferences	Tested concentrations	Results
Ethanol	5, 10, 15, 20, 30, 50%	Interfere at 30%
Ascorbic acid	10, 50, 100, 500, 1000 mg·L^−1^	Interfere at 500 mg·L^−1^
Glucose	10, 50, 100, 500, 1000 mg·L^−1^	Do not interfere at 1000 mg·L^−1^
Fructose	10, 50, 100, 500, 1000 mg·L^−1^	
Sucrose	10, 50, 100, 500, 1000 mg·L^−1^	
Tartaric acid	10, 50, 100, 500, 1000 mg·L^−1^	
Citric acid	10, 50, 100, 500, 1000 mg·L^−1^	
Acetic acid	10, 50, 100, 500, 1000 mg·L^−1^	
Lactic acid	10, 50, 100, 500, 1000 mg·L^−1^	
Tannin	10, 50, 100, 500, 1000 mg·L^−1^	

**Table 3:** Sulfite contents in wines as determined by the proposed method and the Ripper method.

Sample number	Sample type	Sulfite concentration (mg L^−1^)∗	
		Ripper method	Proposed method
1	Sparkling wine 1	24.1 ± 0.2	23.9 ± 0.4
2	Sparkling wine 2	20.3 ± 1.9	19.9 ± 0.2
3	White wine 1	12.9 ± 0.9	12.6 ± 0.7
4	White wine 2	20.6 ± 0.2	20.4 ± 0.5
5	White wine 3	34.7 ± 0.7	34.9 ± 0.3
6	White wine 4	21.5 ± 0.9	22.1 ± 0.2
7	White wine 5	35.5 ± 0.9	34.5 ± 0.3
8	White wine 6	31.7 ± 0.8	30.5 ± 0.1
9	White wine 7	27.3 ± 1.3	23.1 ± 0.3
10	Red wine 1	3.5 ± 0.2	2.28 ± 0.2
11	Red wine 2	8.3 ± 0.1	7.72 ± 0.8
12	Red wine 3	10.6 ± 0.8	10.9 ± 0.2

∗Mean of triplicate results.

## Data Availability

The data used to support the findings of this study are included within the article.

## Supplementary Materials

Supplementary Materials Figure S1: A schematic drawing of a home-made gas diffusion unit for determination of sulfite. Illustration of the zig-zag channel on acrylic block. A photograph of a home-made gas diffusion unit for determination of sulfite. Figure S2: Influence of the sample volume on the response sensitivity. Table S1: Influence of roselle extract concentration on the response sensitivity. Table S2: Influence of the acceptor flow rate on the response sensitivity. Table S3: Influence of the donor flow rate on the response sensitivity.
