# Supplementary material for: An Environmentally Friendly Flow Injection-Gas Diffusion System Using Roselle (Hibiscus sabdariffa L.) Extract as Natural Reagent for the Photometric Determination of Sulfite in Wines
Source: J Anal Methods Chem. 2021 May 18;2021:6665848. doi: 10.1155/2021/6665848 (PMC8154276; doi:10.1155/2021/6665848)
Supplement: Supplementary Materials — Figure S1: A schematic drawing of a home-made gas diffusion unit for determination of sulfite. Illustration of the zig-zag channel on acrylic block. A photograph of a home-made gas diffusion unit for determination of sulfite. Figure S2: Influence of the sample volume on the response sensitivity. Table S1: Influence of roselle extract concentration on the response sensitivity. Table S2: Influence of the acceptor flow rate on the response sensitivity. Table S3: Influence of the donor flow rate on the response sensitivity. [file 6665848.f1.docx]

## **Supplementary Materials**

##
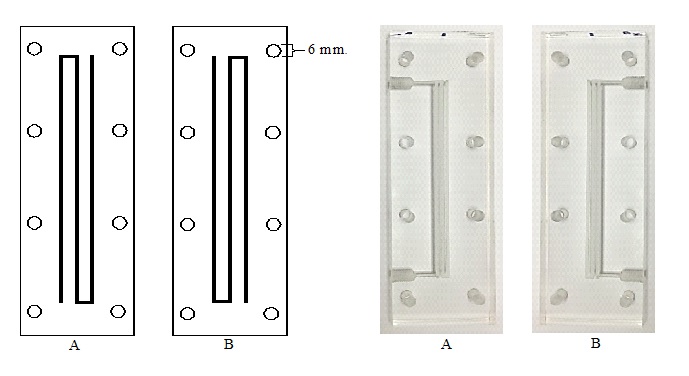

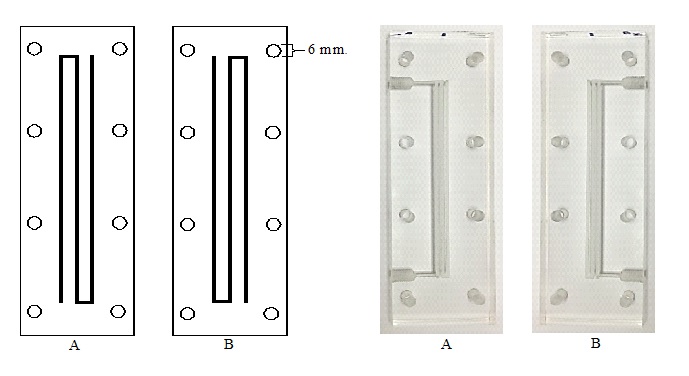
(a) Top view


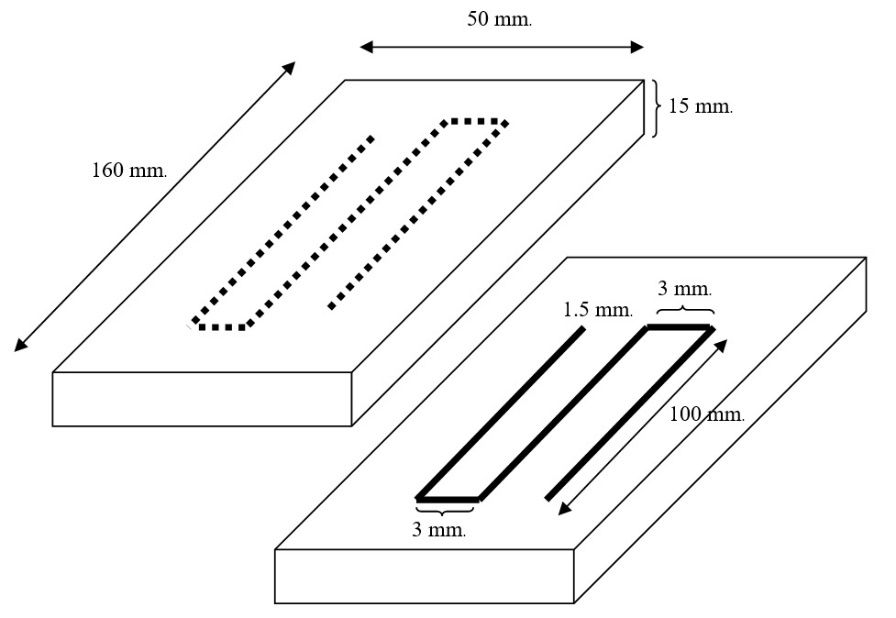
(b) Side view

Figure S1: A schematic drawing of a home-made gas diffusion unit for determination of sulfite. Illustration of the zig-zag channel on acrylic block.


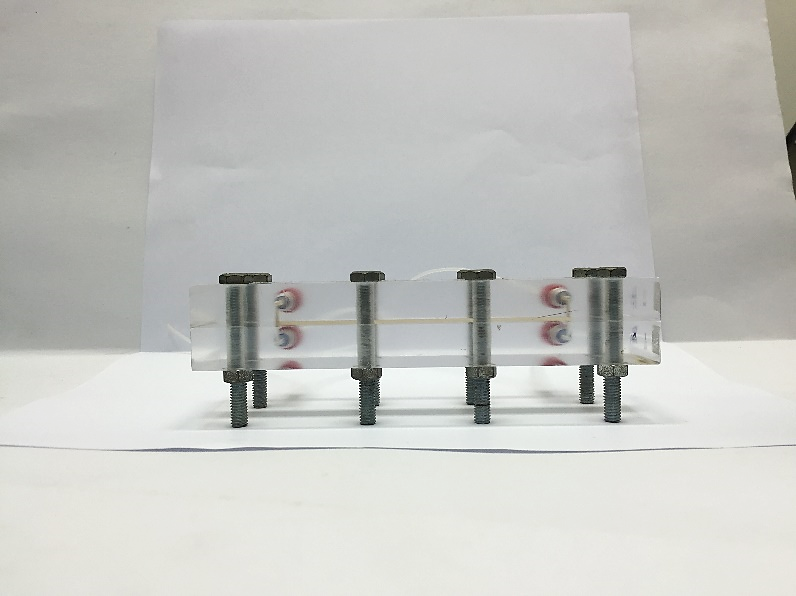
**
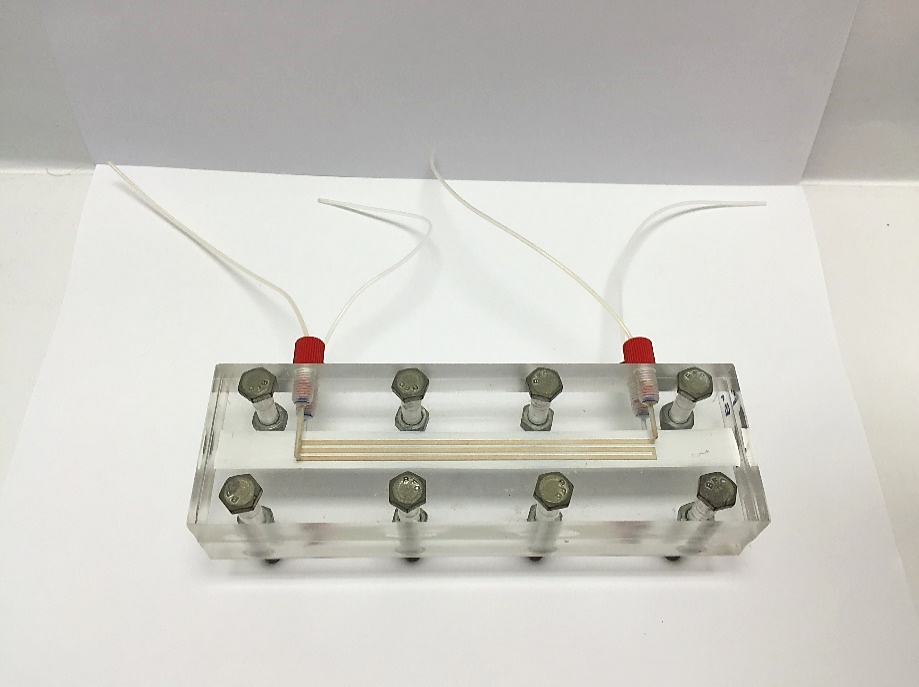
**
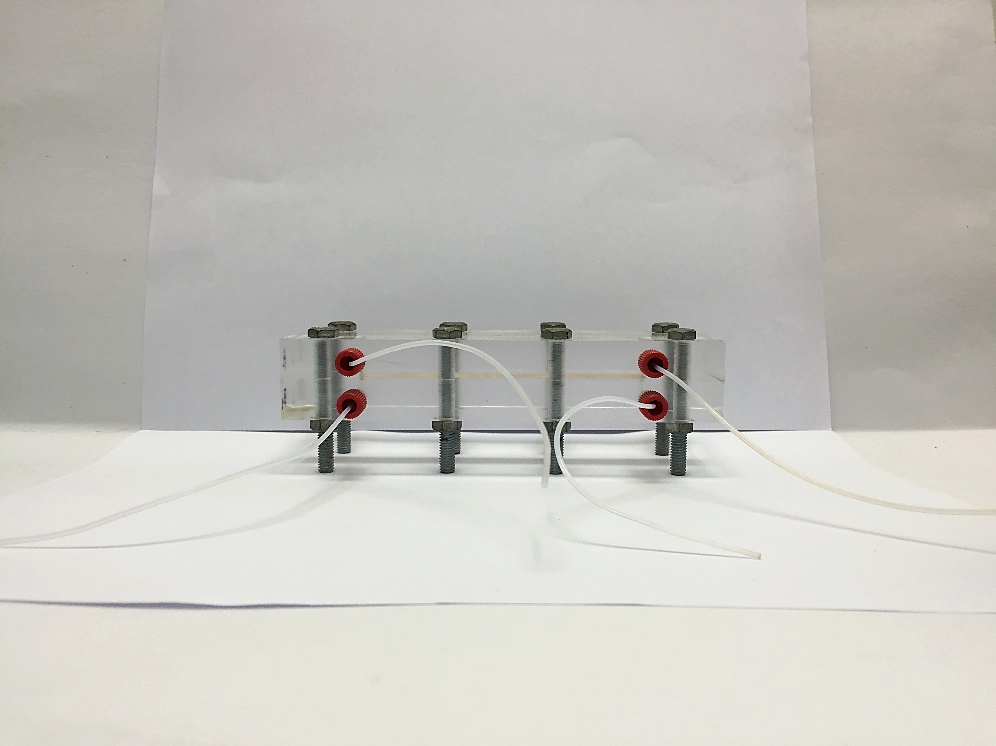


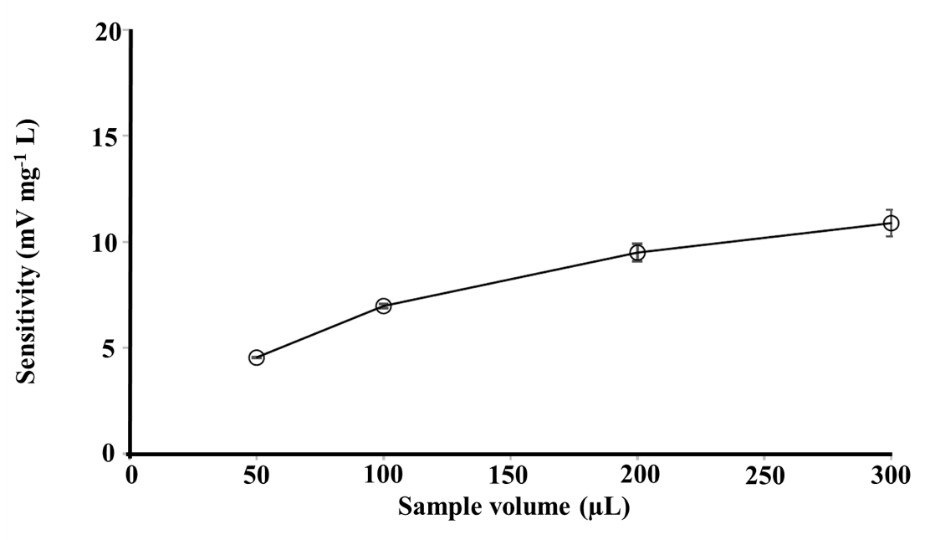
Figure S1: (continued) A photograph of a home-made gas diffusion unit for determination of sulfite.

Figure S2: Influence of the sample volume on the response sensitivity.

Table S1 Influence of roselle extract concentration on the response sensitivity

| Roselle extract concentration  (%w/v) | Linear regression | R^2^ |
| --- | --- | --- |
| 0.6 | y = 0.0080x + 0.0168 | 0.9976 |
| 0.8 | y = 0.0083x + 0.0138 | 0.9984 |
| 1.0 | y = 0.0082x + 0.0124 | 0.9980 |
| 1.2 | y = 0.0078x + 0.0058 | 0.9996 |
| 1.4 | y = 0.0068x + 0.0001 | 0.9998 |
| 1.6 | y = 0.0060x + 0.0016 | 0.9999 |
| 1.8 | y = 0.0054x + 0.0065 | 0.9997 |
| 2.0 | y = 0.0056x + 0.0076 | 0.9996 |
| 2.2 | y = 0.0048x + 0.0078 | 0.9993 |
| 2.4 | y = 0.0043x + 0.0097 | 0.9988 |
| 2.6 | y = 0.0035x + 0.0088 | 0.9986 |

Table S2 Influence of the acceptor flow rate on the response sensitivity.

| Acceptor flow rate (mL min^-1^) | Sensitivity  (mV mg^-1^ L) | R^2^ | Sample throughput (Injection h^-1^) |
| --- | --- | --- | --- |
| 0.5 | 8.6 | 0.9964 | 10 |
| 1.0 | 6.8 | 0.9998 | 18 |
| 1.5 | 5.2 | 0.9995 | 22 |
| 2.0 | 4.1 | 0.9989 | 30 |

Table S3 Influence of the donor flow rate the response sensitivity.

| Donor flow rate (mL min^-1^) | Sensitivity  (mV mg^-1^ L) | R^2^ | Sample throughput (Injection h^-1^) |
| --- | --- | --- | --- |
| 0.5 | 6.8 | 0.9999 | 11 |
| 1.0 | 6.8 | 0.9998 | 18 |
| 1.5 | 6.4 | 0.9999 | 20 |
| 2.0 | 6.0 | 0.9999 | 24 |
